# Methane yield response to pretreatment is dependent on substrate chemical composition: a meta-analysis on anaerobic digestion systems

**DOI:** 10.1038/s41598-024-51603-9

**Published:** 2024-01-12

**Authors:** Thuane Mendes Anacleto, Betina Kozlowsky-Suzuki, Annika Björn, Sepehr Shakeri Yekta, Laura Shizue Moriga Masuda, Vinícius Peruzzi de Oliveira, Alex Enrich-Prast

**Affiliations:** 1 Postgraduate Program in Plant Biotechnology and Bioprocesses, Federal University of Rio de Janeiro, Rio de Janeiro, Brazil; 2 Multiuser Unit of Environmental Analysis, Institute of Biology, Federal University of Rio de Janeiro, Rio de Janeiro, Brazil; 3 Department of Ecology and Marine Resources, Federal University of the State of Rio de Janeiro, Rio de Janeiro, Brazil; 4 Postgraduate Program in Conservation and Ecotourism, Federal University of the State of Rio de Janeiro, Rio de Janeiro, Brazil; 5 Postgraduate Program in Neotropical Biodiversity, Federal University of the State of Rio de Janeiro, Rio de Janeiro, Brazil; 6 Department of Thematic Studies-Environmental Change, Linköping University, Linköping, Sweden; 7 Biogas Solutions Research Center, Linköping University, Linköping, Sweden; 8 Chico Mendes Institute for Biodiversity Conservation-ICMBio, Brasilia, Brazil; 9 Institute of Marine Science, Federal University of São Paulo (IMar/UNIFESP), Santos, Brazil

**Keywords:** Biotechnology, Environmental sciences, Energy science and technology

## Abstract

Proper pretreatment of organic residues prior to anaerobic digestion (AD) can maximize global biogas production from varying sources without increasing the amount of digestate, contributing to global decarbonization goals. However, the efficiency of pretreatments applied on varying organic streams is poorly assessed. Thus, we performed a meta-analysis on AD studies to evaluate the efficiencies of pretreatments with respect to biogas production measured as methane yield. Based on 1374 observations our analysis shows that pretreatment efficiency is dependent on substrate chemical dominance. Grouping substrates by chemical composition e.g., lignocellulosic-, protein- and lipid-rich dominance helps to highlight the appropriate choice of pretreatment that supports maximum substrate degradation and more efficient conversion to biogas. Methane yield can undergo an impactful increase compared to untreated controls if proper pretreatment of substrates of a given chemical dominance is applied. Non-significant or even adverse effects on AD are, however, observed when the substrate chemical dominance is disregarded.

## Introduction

Anaerobic digestion (AD) is a successful and robust waste treatment biotechnology converting organic waste into clean energy in the form of biogas^1^ and recovering nutrients as fertilizers and soil conditioners^2^. AD plays a crucial role in achieving the ambitious goal of the European Climate Law, aiming for climate neutrality by 2050^2^. An estimated increase from 0.3 EJ to 8.3 EJ by 2050 from biogas upgraded to biomethane (90% methane) makes it the non-fossil source with the greatest potential to be carbon neutral^2^. AD systems mitigate the emission of greenhouse gases (GHG), by recovering methane (CH_4_) from organic wastes, and, when used as a combustion fuel, release carbon–neutral carbon dioxide (CO_2_)^3^. About 60 to 80% of GHG emissions from transportation can be reduced by replacing gasoline with biomethane produced from AD^4^. Currently, the global potential for energy generation from biogas is estimated to be 10,000 to 14,000 TWh, with the potential to replace up to 10% of the world's primary energy consumption^5^ of electric power, heat and automotive fuel. Unlike other sources of non-fossil energy, organic residues are the raw primary source for biogas production, which is relatively less sensitive to seasonality or scarcity.

Due to integrated socioenvironmental benefits^1^ e.g., the replacement of energy resources such as firewood by biogas can improve quality of life, and promote gender equality, and higher educational levels^6^. AD surpasses several other renewable energy sources^7^ representing the major technological pathway for the implementation of the United Nations Sustainable Development Goals (SDGs)^4^. Besides expanding local employment opportunities^6^, AD promotes energy decentralization, with electricity supply to remote areas, e.g., rural communities by the implementation of small-scale biogas plants or by direct injection into the existing natural gas grid^4,8,9^.

AD follows 4 steps: hydrolysis, acidogenesis, acetogenesis and methanogenesis^9^. Hydrolysis by microbial extracellular enzymes converts complex biopolymers (i.e., protein, lipid, polysaccharides) into smaller compounds (i.e., sugar, amino acids, fatty acids)^10^, which in turn are converted into volatile fatty acids (VFA), CO_2_ and H_2_ in the acidogenesis step^11^. Subsequently, acetate is produced in the acetogenesis step, providing the product for the generation of mainly CH_4_ and CO_2_ in the methanogenesis step^10,11^. Studies have exhaustively identified hydrolysis as the bottleneck for biogas production from recalcitrant biomass^12,13^ usually leading to low AD efficiency upon application in, for example, agricultural sectors^14^.

Substrates are often subjected to pretreatment prior to AD, and the potential of pretreatments to improve hydrolysis has been extensively reported in the literature. Several chemical, physical and biological pretreatments (Fig. S1) are applied to organic wastes to modify their physical–chemical structures and improve their biodegradability^15–17^. The resulting reduction in particle size and increase in surface area, porosity, and solubility of particulate organic matter^18^ enhances the accessibility by microorganisms, improving hydrolysis and biogas production^19^. However, all of those pretreatments also increase the cost of the AD process, as they lead to increased energy consumption, require the purchase of additives, and usually depend on operational investments to adapt equipment to suit the pretreatment^13,20^. In addition, pretreatments may even have adverse effects on AD and result in lower CH_4_ yields^1,21^ if the selected pretreatment is not suitable for a given substrate. The proper choice of pretreatment is crucial to achieving viable and cost-effective conversion of recalcitrant feedstocks and to increasing biogas production^20^; therefore, the effects of pretreatment on organic wastes must be evaluated with respect to the chemical composition of the biomass.

Grouping substrates by origin (e.g., agricultural, municipal, industrial wastes, and aquatic biomass) is a widespread and common strategy applied in the industry to lower logistics costs and to promote the digestion of the greatest amount of waste available in a given geographic area. This has led to the application of pretreatments disregarding the heterogeneity of the biomass chemical composition or even to the implementation of co-digestion. Co-digestion is a strategy applied for simultaneous management of different waste streams by AD where two or more types of feedstock are combined^22^. Since in co-digestion the substrate is mixed as a strategy to optimize the AD process^9,22^ (e.g. balancing macro and micronutrients supply, and the moisture content or diluting inhibitory compounds), interventions such as pretreatment may lead to adverse process performance due to organic matter overload. For instance, co-digestion of (30% primary sludge and 70% sewage sludge) and glycerol (1% v/v) decreased CH_4_ yields from 500 to 70 mL/gVS_added_ after alkaline pretreatment application^9^. Several studies (e.g.^15,17,23–25^) have tested the application of specific pretreatments to specific substrates, but to the best of our knowledge, not a single study has yet consistently quantified the efficiencies of different pretreatments with varying types of substrates sorted by predominant chemical composition. Identifying proper pretreatments by substrate chemical predominance may open an opportunity for the management of new organic streams (individual or in combination) via AD. Also, it prevents unnecessary costs as the pretreatment implementation comprises a substantial proportion (up to ca 20%) of the total biomethane production cost^26^.

Here we conducted a systematic review and a comprehensive meta-analysis to quantify the performance of different pretreatments according to the predominant chemical composition of the organic waste. Despite inherent limitations of performing a meta-analysis in AD systems, e.g., encompassing variations in operating conditions and feedstock characteristics across studies, the application of meta-analysis in AD systems offers substantial advantages. The outcomes derived from meta-analysis play a pivotal role in steering research efforts, shaping best practices, and advancing the knowledge base in AD systems. A comprehensive synthesis of the existing research allows for the identification of trends and overarching insights that may not be apparent in individual studies. Here, we evaluated 192 studies from which 1374 individual effect sizes were calculated from peer-reviewed scientific articles over the past 45 years (Table S1) and provide a comprehensive decision-making guideline for the choice of appropriate pretreatment based on the predominant organic chemical composition of the substrates.

## Substrate chemical composition affects pretreatment efficiency

**Figure 1:**
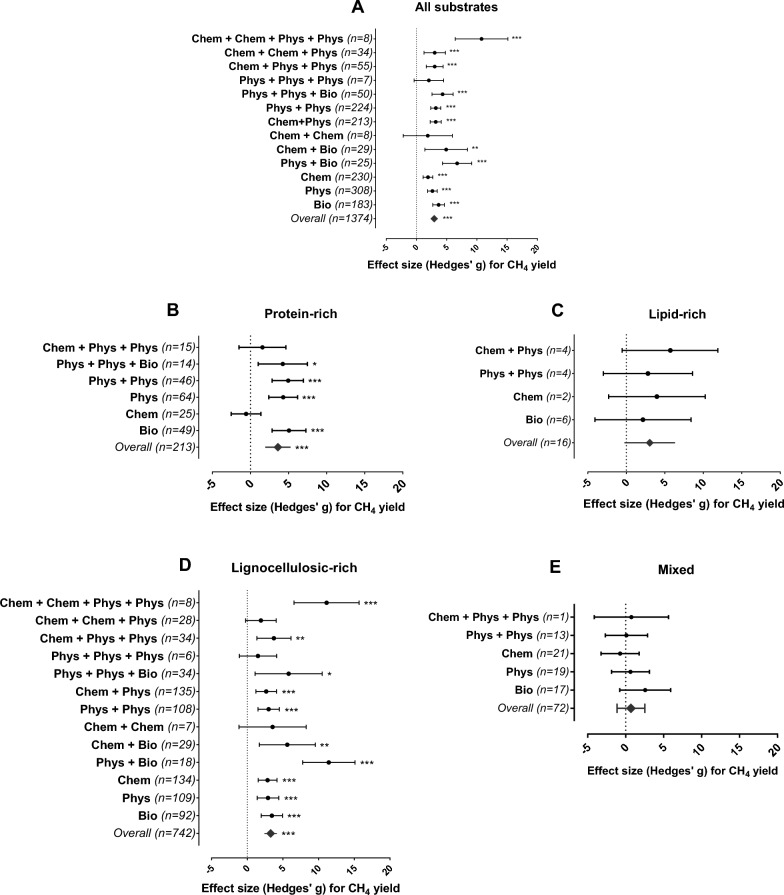
Mean effect size (Hedges’ g) and 95% confidence intervals for CH_4_ yield from protein-, lipid- and lignocellulosic-rich substrates subjected to different pretreatments. *Phys* physical, *Chem* chemical, *Bio* biological (Figs. S3–S5); these abbreviations denote the treatments and combinations applied to different substrates. (**A**) All substrates were sorted by pretreatment regardless of their chemical composition. (**B**) Protein-rich substrates were predominantly composed of animal waste, microalgae, or high protein content (≥ 40% dry matter). (**C**) Lipid-rich substrates were predominantly composed of agricultural oil residues, swine slaughterhouse wastewater, or any source with high lipid content (≥ 40% dry matter). (**D**) Lignocellulosic-rich substrates were predominantly composed of crop residues, cattle manure, or high lignocellulose content (≥ 50% dry matter). (**E**) Mixed substrates included only food waste. Detailed information on the substrate categories can be found in the Supplementary material (Figs. S6–S8). Significance level: p ≤ 0.001 (***); p ≤ 0.01 (**); p ≤ 0.05 (*). n = number of effect sizes per treatment type.

The effect and magnitude of the different pretreatments were assessed by calculating the standardized mean difference (SMD), which is the CH_4_ yield difference between the treated and untreated (control) substrate groups. SMD Hedges’ g ≤ 0.2 represents a small effect, 0.3–0.5 a medium effect, and ≥ 0.6 a large effect^27^. CH_4_ yield is significantly improved by a given pretreatment if SMD is higher than zero and the lower limit of the confidence interval (CI) does not cross zero, while significantly depressed by a given pretreatment if SMD is lower than zero and the upper limit of the CI does not cross zero. Our findings indicate that to reach higher efficiencies for biogas production, classification based on chemical predominance rather than on the origin of the waste, prior to the choice of proper pretreatment is fundamental (Fig. 1).

## Protein-rich substrates

About 1 million tons of protein-rich waste is produced globally every year^12^. Although protein-rich substrates have high theoretical methane potential, ca 0.5 Nm^3^/kg volatile solid (VS), AD can be severely affected by ammonia accumulation from protein breakdown^12,28^. High concentrations of ammonia can particularly inhibit acetoclastic methanogenesis^18^, leading to VFA accumulation, a lower biomethane yield, and process disturbances^3^.

Our literature search demonstrated that microalgae, meat processing waste, slaughterhouse waste, and swine and chicken manure are those substrates that have been reported as protein-rich feedstock of AD^29^. Microalgae were the most common feedstock studied among protein-rich substrates (Fig. S6), which can be explained by their rapid growth rates and cultivation viability without requiring arable lands^16^.

**Figure 2:**
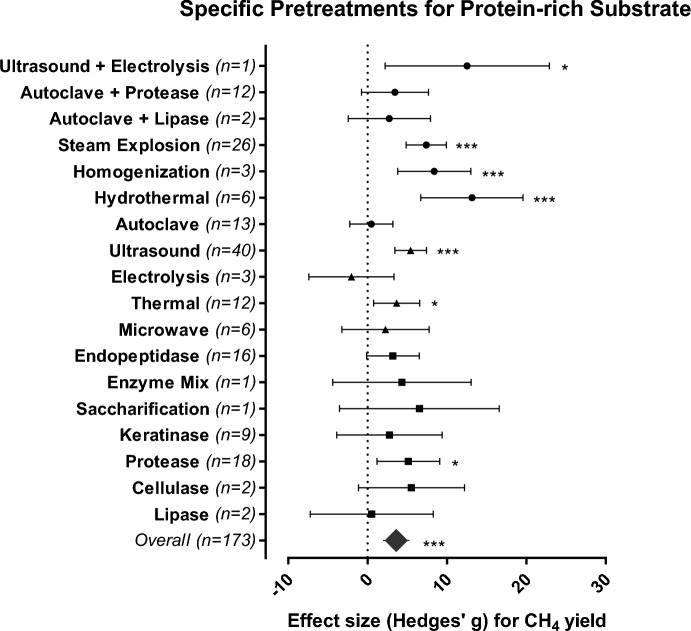
Mean effect size (Hedges’ g) and 95% confidence intervals for CH_4_ yield for the most efficient pretreatment methods (biological = squares, physical = triangles and combinations thereof = circles) applied to protein-rich substrates; the plot depicts 95% confidence intervals of the Hedges’ g effect size for CH_4_ yield. Significance level: p ≤ 0.001 (***); p ≤ 0.01 (**); p ≤ 0.05 (*). n = number of effect sizes per treatment type.

The outcomes of the meta-analysis resulted in 213 effect sizes from pretreatment of protein-rich substrates (Fig. 1B). Biological (SMD = 5.061, 95% CI 2.839–7.282) and physical (SMD = 4.301, 95% CI 2.405–6.197) pretreatments applied alone or in combination led to the highest CH_4_ yields from protein-rich substrates (Fig. 1B), while chemical pretreatments (SMD = − 0.573, 95% CI − 2.520 to 1.374) had no significant effect. Biological pretreatments (e.g., enzymatic pretreatment), which increase protein hydrolysis and solubilization^16^. Some biological pretreatments such as bacteria flocculation (flocs) increase methanogens tolerance to NH_3_ concentration and toxic compounds (i.e., furfural)^12^. At full-scale, biological pretreatments have proven to further reduce substrate viscosity and the energy demand for mixing^30^. In particular, the application of protease as enzymatic pretreatment led to a significant increase in CH_4_ yield (SMD = 5.132, 95% CI 1.178–9.085, Fig. 2), which can be attributed to the specificity of protease in hydrolyzing proteins. The application of protease is associated with low pollution risk to the environment and low energy demand, making it more suitable than energy-intensive options such as thermal pretreatments at the laboratory or full-scale^30^. The overall advantages of biological pretreatments are their reaction specificity (in case of enzymatic pretreatment), low operating and energy costs, and a lack of toxic end products^15^.

Pretreatments that involve heat application, including thermal (SMD = 3.655, 95% CI 0.748–6.561), steam explosion (SMD = 7.386, 95% CI 4.851–9.922), and hydrothermal (SMD = 13.144, 95% CI 6.693–19.595) were those exhibiting the best performance for protein-rich substrates (Fig. 2). These pretreatments are effective in breaking down organic matter and increasing its exposure to enzymatic degradation during the hydrolysis step^19^. Heat pretreatments are one of the most applied in full-scale biogas plants^9^, which may be a result of the mandatory pasteurization requirement for some substrates. However, the relatively high cost:effectiveness ratio of these pretreatments discourages their use, especially when compared with biological pretreatments, which are relatively inexpensive to implement.

Homogenization is a promising physical pretreatment at the industrial scale, as it disrupts substrate structure and decreases particle sizes, consequently improving the substrate accessibility for microbial degradation^23^. Homogenization significantly increased the CH_4_ yield (SMD = 8.339, 95% CI 3.798–13.001) of protein-rich substrates. Similarly, ultrasonication (SMD = 5.421, 95% CI 3.434–7.407, Fig. 2) promotes organic waste degradation via hydromechanical stress, reducing hydrolysis time and increasing the production of biogas^17^. Although homogenization requires high pressure (> 800 bar) to increase up to 15% of the protein solubilization, the energy balance of the pretreatment is positive^9^, as energy costs are covered by biomethane production, and has been successfully applied on a full-scale^23^. Ultrasonication is equally successful at practical levels, producing 3–10 kW in CH_4_ yield for every kilowatt of ultrasonic energy applied^17^.

Chemical pretreatments applied to protein-rich substrates led to an overall reduction, though non-significant, in CH_4_ yield (Fig. 1B). This can be attributed to the generation of secondary degradation products from complex molecular bonds of proteins in addition to the formation of inhibitory compounds such as ammonia^12^.

## Lipid-rich substrates

Milk and meat processing waste, oilseeds, and kitchen waste are examples of lipid-rich substrates (Fig. S7)^31^. Lipid-rich substrates can exhibit greater biogas production than protein- and carbohydrate-rich substrates^32^, with the theoretical methane potential of ca 1.0 Nm^3^/kg VS^10^. Lipids consist of long-chain fatty acids (LCFAs) linked to glycerol, alcohols or other groups by ester or ether linkages^31^. However, high concentrations of LCFAs are harmful to AD and cause severe inhibition to microorganisms, especially in the acetogenesis and methanogen stages^31^.

**Figure 3:**
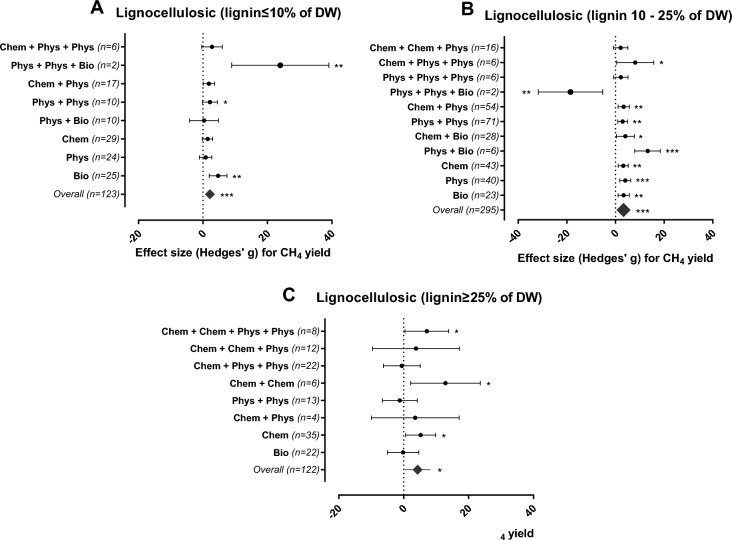
Mean effect size (Hedges’ g) and 95% confidence intervals of CH_4_ yield for lignocellulosic-rich substrates subjected to different pretreatments. *Phys* physical, *Chem* chemical and *Bio* biological; these abbreviations denote the treatments and their combinations applied to substrates with different lignin contents. (**A**) lignin < 10%, (**B**) lignin 10–25% and (**C**) lignin > 25% DW. Significance level: p ≤ 0.001 (***); p ≤ 0.01 (**); p ≤ 0.05 (*). n = number of effect sizes per treatment type.

As shown in Fig. 1C, 16 effect sizes were calculated for lipid-rich substrates. Pretreatments had marginal positive effects, and none of the tested categories yielded a higher efficiency than those of the non-pretreated controls (Fig. 3C). However, this result should be interpreted with caution, as the number of observations was considerably lower than the number reported for other substrates.

The use of lipid sources as a sole substrate is not a common practice for biogas production due to the need for nutrient balance (C:N:P:S ratio) to achieve optimal microbial activity. Thus, substrates with high lipid content (> 60% of wet weight) achieve the highest production of biogas in co-digestion^33^. Nevertheless, biogas production can be hampered by excessive loads of lipids due to the hydrophobic nature of lipid-rich materials^32^ and by disturbances such as foaming that inhibit microbial activity^31^.

Appropriate pretreatment can mitigate the AD instability associated with high loads of waste lipids by improving the dispersion and solubilization of lipids in the sludge matrix^19^. Nevertheless, our results suggest that optimizing the balance of substrates and nutrient ratios via co-digestion could be more promising than investments in pretreatments. LCFAs from the lipid-rich substrate are usually stabilized when co-digested with low biodegradability co-substrates^10^, improving overall biogas production. Alternative operational approaches such as effluent solid recirculation or pulse feeding has also shown promising results on increasing the capacity of AD for handling high loads of lipids^34,35^.

## Lignocellulosic-rich substrates

Lignocellulosic biomass is one of the most abundant sources globally for biofuel production^20^. Approximately 181.5 billion tons of lignocellulosic biomass are generated worldwide every year^36^. It is classified by its molecular organization consisting of crystalline cellulose, organized into macrofibrils firmly attached by intermolecular hydrogen bonds, combined with amorphous chains of hemicelluloses, all immersed in a lignin matrix^37^. However, the broad chemical heterogeneity of this organic source prevents the application of a single operational condition that meets all requirements of this feedstock^38^. The biogas production of its widely heterogeneous composition decreases dramatically if treated under equal operating conditions^38^. Although feedstocks e.g., hardwoods, soybeans, sugar beets, manure, and sugarcane bagasse have been treated under the same classification, their distinct content of biopolymers sorts them apart.

A total of 742 effect sizes were calculated for lignocellulosic substrate, more than the sum of all other substrates (Fig. 1D). With a few exceptions, pretreatments applied to lignocellulosic-rich biomasses had positive effects on CH_4_ yields, despite an unclear response towards specific pretreatments (Fig. 1D). This was probably a result of a large number of different biomass sources that were merged into this group implying large variations in the substrate chemical composition. Lignocellulosic biomass e.g., wood, energy crop, and plant residues are primarily comprised of cellulose, hemicellulose, and lignin, and the composition of these components determines the recalcitrance nature and biodegradability of their chemical structure^25,37^.

Lignin in plants mainly provides structural support, impermeability, and resistance against microbial attack and oxidative stress^25^. Despite the difficulty in degrading lignin, the application of appropriate pretreatment resulted in a CH_4_ yield increase of almost 40%^39^. Lignin content has been identified as one of the main barriers to the AD of lignocellulosic biomass^11^ and can be used as an independent variable to assess the effects of pretreatments on lignocellulosic-rich substrates^14^. Therefore, lignocellulosic-rich substrates were divided into three categories according to their lignin content (< 10%, 10–25%, and > 25% lignin dry weight (DW), Fig. 3).

Chemical pretreatments degrade lignin very efficiently and are commonly applied to overcome the recalcitrance of lignocellulosic-rich organic residues^26^. Chemical additives (such as sulfuric acid, hydrochloric acid, sodium hydroxide, potassium hydroxide, lime, and hydrogen peroxide) remove the protective barrier created by lignocellulosic fibers, increasing cellulose exposure and facilitating its degradation during AD^26^. However, chemical addition implies an increase in operational costs when applied at full-scale^40^ related to chemical reagents and construction of corrosion-resistant reactors^41^. Generation of toxic compounds^4^ that can disturb biogas production is also identified as a drawback of using chemical pretreatments^4^. Nevertheless, the overall effect of various chemical pretreatment applied on lignocellulosic-rich substrates resulted in an increase in CH_4_ yield based on the outcomes of our meta-analysis (Fig. 3A–C).

Interestingly, at low and medium lignin content (< 25% lignin DW), combined physical and biological pretreatments were more efficient than the addition of chemicals and should be used preferentially if the main reason for pretreatment is to increase CH_4_ yield. As an exception, biogas production from the lignocellulosic substrate at medium lignin content (Fig. 3B), dropped dramatically when subjected to a combination of temperature, pressure and enzymatic pretreatment, in contrast to the high performance of the physical + biological combination^9^. The adverse effect possibly occurred in response to multiple interventions generating a highly bioavailable organic matter, overloading the AD system negatively affecting biogas production^9^.

**Figure 4:**
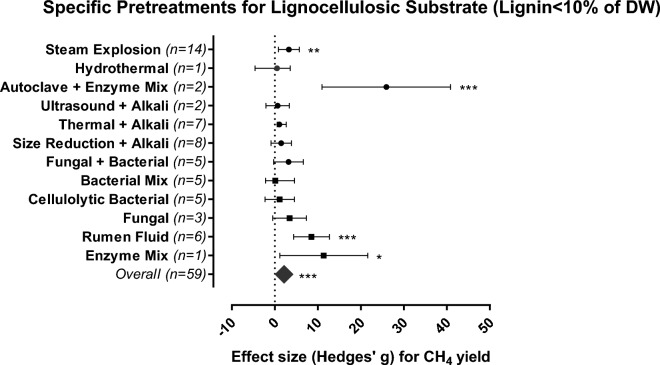
Methane yields for the most efficient pretreatment methods (biological = squares, combinations = circles) applied to lignocellulosic-rich substrates (lignin < 10% DW). The plots depict 95% confidence intervals of Hedges’ g effect size for CH_4_ yield. Significance level: p ≤ 0.001 (***); p ≤ 0.01 (**); p ≤ 0.05 (*). n = number of effect sizes per treatment type.

Lignocellulosic substrates with low lignin contents (≤ 10% DW) have less of a protective barrier and are therefore more susceptible to biodegradation; hence, pretreatment may have no effect or even an inhibitory effect on CH_4_ yield due to the accumulation of toxic compounds such as phenolic substances, 5-hydroxymethylfurfural (HMF) furfurals and aldehydes^1,42^. Our results suggest that substrates with low lignin content require only milder interventions, including the application of biological pretreatments, e.g., enzymes. Enzymatic pretreatment alone (SMD = 11.390, 95% CI 1.169–21.610) or combined with autoclavation (SMD = 25.941, 95% CI 10.998–40.884) or rumen fluid addition (SMD = 8.525, 95% CI 4.368–12.682) led to the highest CH_4_ yields from substrates at low lignin content (Fig. 4). Up to 83% increase in CH_4_ yields of low-lignin substrates was achieved after biological pretreatment (Table S3).

Sugar beet pulp and Napier grass are examples of lignocellulosic sources with low lignin content that were subjected to biological pretreatment (Table S2; Fig. 4). The addition of microbial consortia (bacteria and fungi) and enzymes for pretreatment, not only preserved the weight of cellulose for the hydrolysis step but also increased ca 84% of the total sugar yield which serves as methanogenic substrate in AD systems^43^. Also, enzymes from fungi have been reported as a strategy for the optimization of AD on full-scale, where its addition increased CH_4_ yield by 8% and reduced the AD operational costs by 10%^30^. Thus, indicating that, the use of biological pretreatments of lignocellulosic substrates with lignin content < 10% should be prioritized over the use of chemicals.

**Figure 5:**
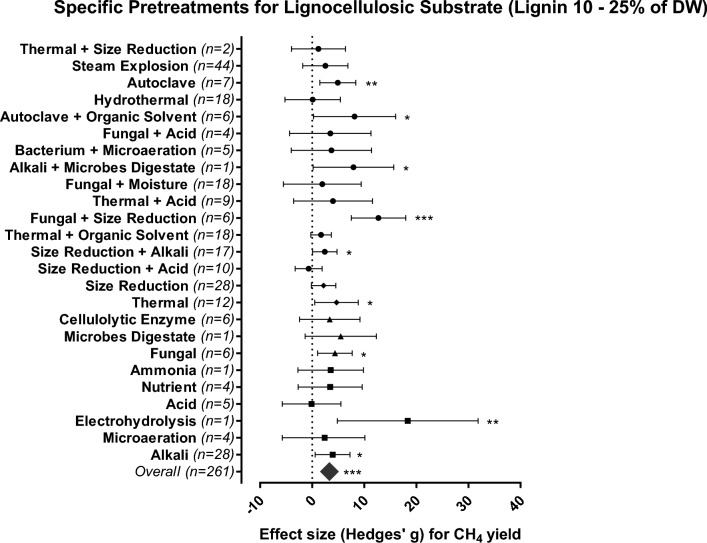
Methane yield effects for the most efficient pretreatment methods (chemical = squares, biological = triangles and combined methods = circles) applied to lignocellulosic-rich substrates (lignin 10–25% DW). The plots depict 95% confidence intervals of Hedges’ g effect size for CH_4_ yield. Significance level: p ≤ 0.001 (***); p ≤ 0.01 (**); p ≤ 0.05 (*). n = number of effect sizes per treatment type.

Most agricultural residues have intermediate levels of lignin content (10–25% DW)^39^ and comprised the majority of the lignocellulosic substrates used for biogas production (Fig. 3) with 295 individual effect sizes. The overall effect of all pretreatments applied to lignocellulosic substrates with intermediate lignin contents was positive and significant (SMD = 3.331, 95% CI 2.055–4.607, Fig. 5).

A common strategy used in the agricultural sector to deal with intermediate lignin content is to apply physical pretreatment to reduce particle sizes; this process alone has a small positive effect. However, combining particle size reduction with fungal (SMD = 12.734, 95% CI 7.520–17.948) or alkaline (SMD = 2.426, 95% CI 0.082–4.771) addition significantly enhanced CH_4_ yields (Fig. 5) and led to increases of up to 170% compared to the untreated substrate (Table S4). Particle size reduction increases surface area and facilitates microbial access to biodegradable cellular compounds^13^; furthermore, when this approach was combined with the application of ligninolytic enzymes excreted by fungi, a highly delignified biomass was obtained, and the benefits of this combined approach surpassed the positive effect of fungal addition alone (SMD = 4.377, 95% CI 1.050–7.703, Fig. 5).

Alkaline addition decreases the recalcitrance of lignocellulosic materials by enhancing lignin and hemicellulose solubilization, thus reducing the crystallinity of the cellulose^37^. It also promotes the removal of acetyl groups and uronic acid substitutions in hemicelluloses, increasing access to carbohydrates during hydrolysis, being more favorable for biomass with low/medium lignin content^44^. Alkaline pretreatments alone had positive effects (SMD = 3.936, 95% CI 0.594–7.277) on CH_4_ yield and can be considered for application as the only pretreatment since this approach is cost-effective even at full-scale^13^.

Thermal (SMD = 4.675, 95% CI 0.498–8.852) and autoclave (SMD = 4.920, 95% CI 1.468–8.372) are physical pretreatments that resulted in significant increases in CH_4_ yields when applied to substrates with moderate lignin contents. The increase in temperature promotes cell lysis making intracellular material available for microbiological degradation^41^. Autoclaving is a combined pretreatment method involving high temperatures and pressures and leads to a steam explosion when applied to organic matter.

**Figure 6:**
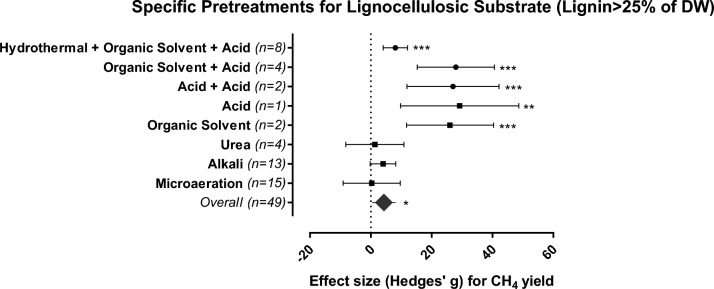
Methane yield effects for the most efficient pretreatment methods (chemical = squares and combinations = circles) applied to lignocellulosic-rich substrates (lignin > 25% DW). The plot depicts 95% confidence intervals of Hedges’ g effect size for CH_4_ yield. Significance level: p ≤ 0.001 (***); p ≤ 0.01 (**); p ≤ 0.05 (*). n = number of effect sizes per treatment type.

The lignin content in lignocellulosic-rich substrates is proportional to the ability of the substrate to withstand microbial hydrolysis^13^. Accordingly, lignocellulose substrates with lignin contents above 25% e.g., woods, stalks, processed bagasse, and silage (Fig. S8) are less effectively biodegraded and exhibit limited potential for methane production. Substrates with this high lignin content have been more rarely tested leading to only 122 individual effect sizes (Fig. 6), for which chemical pretreatments applied alone or in combination are the only viable strategy for increasing the CH_4_ yield.

Acid pretreatments are the most commonly applied to such substrates with a CH_4_ yield increase in up to 500% (Table S5). The addition of acid can accelerate the sugar conversion rate over 90%, by promoting the breakdown of glycosidic bonds of long chains of cellulose and hemicellulose into sugar monomers^44^. However, the use of acids requires extra care, as high concentrations of reagents can cause serious damage and corrosion of the operational system in addition to causing imbalances in the AD process^38^. At a practical level, chemical addition handled with accuracy and caution is supported techno-economically^13^ despite the requirement of high investments for operation and final safe environmental disposal via the digestate^11^.

## Mixed substrates

As mentioned earlier, substrate mixing is a very common practice, either to treat all organic waste from a given location in a single operation or to perform co-digestion. However, except for co-digestion, chemical predominance and nutrient balance are not often considered for mixed substrates. Here, mixed substrates are those in which carbohydrates, lipids, and proteins are roughly equal without major disproportions between their contents. Although food waste, sewage, and co-digestion comprise a mixture of several organic sources, food waste seems to be the most suitable to be used as a model, since co-digestion prioritizes geographical location and stabilization of organic matter without the addition of pretreatment^45^ while the sewage is often pointed out as lipid-rich^19^.

Food waste constitutes a complex organic matrix where the final composition depends on eating habits and varies between countries, regions and periods of the year^46^, preventing a unified characterization of food wastes. From the 72 individual estimated effect sizes, there were no significant differences among pretreatments applied to food waste with an overall effect of SMD = 0.693, 95% CI − 1.132 to 2.518 (Fig. 1E). The outcomes highlight that the application of pretreatments might even have a negative marginal effect on CH_4_ yield of food waste. Therefore, the appropriate pretreatment should be identified on a case-by-case basis depending on the chemical predominance of the analyzed substrate^47^. If no chemical component predominates, targeted pretreatment cannot be advised, and therefore, positive effects on substrate degradation might be drastically reduced. Therefore, the selection of pretreatments applied to mixed substrates with undefined chemical compositions should consider other factors, such as decreased costs or the need to meet legal requirements (i.e., pasteurization).

## Conclusions

Lack of cost-effective pretreatment options or the application of suboptimum pretreatments to specific substrates are among the factors that currently limit the global potential for biogas production. Our meta-analysis showed that the choice of pretreatment should be defined by the predominant chemical composition of the targeted organic waste. For example, major global crop residues including corncob, rice husk, rice straw, sugarcane bagasse, and wheat straw with a combined annual generation of ca 1.3 billion tones by the key producing countries are all grouped as lignocellulosic substances with intermediate lignin content based on our categorization (< 25% lignin). Most of the studies (87%) utilize laboratory batch conditions using a Biochemical Methane Potential (BMP) assay for pretreatment evaluation. Despite concerns of upscaling results to the industry, BMP assays are the first step applied by researchers and industrial biomethane producers for the evaluation of the feasibility of biomass as a feedstock for AD. Thus, the outcomes reported based on BMP quantifications can aid the selection of suitable pretreatments for laboratory- or pilot-scale simulations of AD processes for the industry. Our outcomes suggest that the current methane potential of these substrates could be enhanced by up to 170% if appropriate pretreatment methods are applied. This would add up to 1800 TWh of the global renewable energy potential assuming roughly 90% dry matter content and a conservative methane potential of 220 m^3^ CH_4_ per dry weight of the untreated feedstock. The guideline provided in this study assists selection of proper pretreatment methods based on the knowledge generated in past 45 years to boost economic gains and promote the contribution of AD to societal sustainability and decarbonization.

## Methods

### Search strategy and study selection

We performed a systematic review and meta-analysis of studies published in the Web of Science database between 1975 and July 2020 based on the Preferred Reporting Items for Systematic Reviews and Meta-Analyses (PRISMA, http://www.prisma-statement.org/) checklist. The search was performed using the following keywords: “hydrolysis”, “anaerobic digestion”, “methane yield” and “pretreatment”*.* The search was restricted to only articles (document type) and only publications in English (language) (Fig. S9).

The eligibility criteria for inclusion of articles in the meta-analysis were as follows: (i) description of the average value, standard deviation (SD) and number of replicates for methane yield with and without pretreatment (control); (ii) description of the pretreatment applied; and (iii) methane yield provided separately from the total biogas production rate. We included studies with replicates ranging from 2 to 5, recognizing that, despite the general recommendation of a minimum of 3 replicates for Biochemical Methane Potential (BMP) tests, particularly for treatment bottles, the number of replicates of larger lab-scale reactors are seldom above 2.

### Data collection

Articles eligible after screening by the inclusion criteria had their data collected in an Excel spreadsheet. The data extracted from each article includes general information (e.g., first author's name, article title and year of publication), substrate type, substrate chemical composition, inoculum description, operational configuration (e.g., temperature condition, hydraulic retention time (HRT), stirring (i.e., RPM), reactor type, operational scale, total volume and working volume), pretreatment method, specific pretreatment conditions and methane yield data (mean, standard deviation (SD) and number of replicates).

Pretreatment techniques (e.g., autoclave, mechanical, alkaline, acid and enzyme) were grouped into methods (e.g., physical, chemical, biological and combined) since the transformations achieved in organic matter are rather similar within techniques belonging to the same group^26^. Once the effect of each pretreatment method is significant in the quantitative synthesis, all the techniques that compose it are individually evaluated. Also, the different feedstocks were grouped by the predominance of the chemical composition.

### Substrate classification by predominant chemical composition

The substrates tested in the studies included in the meta-analysis were grouped into categories according to their predominant chemical composition in dry weight (DW). Based on the chemical characterization reported in the articles from the systematic review, the substrates were divided into 4 main categories: protein-rich, lipid-rich, lignocellulosic-rich and mixed.

As the AD literature does not present a range of protein content for protein-rich substrates^12,30,32^, data from the articles included in the systematic review were screened in order to assess their chemical composition. Protein-rich substrates were then considered those with an average protein content of ≥ 40% DW.

Due to operational limitations mono-digestion of lipid-rich substrates is rare^32,33^, and so is the chemical characterization. Based on the classification of lipid-rich substrates from previous studies in the literature, the average lipid content of lipid-rich substrates was ≥ 40% DW.

Lignocellulosic substrates have at least > 50% lignocellulose content per DW. The chemical composition of lignocellulosic biomass is composed of three main biopolymers: cellulose, hemicellulose and lignin^39^. Lignin was selected as the independent variable due to its widespread description in the literature as one of the main barriers to the degradation of lignocellulosic content^11^. Lignocellulosic substrates were here divided into three lignin concentration ranges. The choice of lignin content range was based on the difficulty in converting crop residues into biogas in the range of 10–25% DW of lignin applied as mono-digestion, either due to the complexity of the structure of the material or the generation of phenolic compounds that inhibit AD^36^. In addition, most crop residues applied to energy generation are in this range of lignin content, which requires high attention to optimize the digestion^36^. Lignocellulosic substrates were then classified into 0–10%, 10–25% and > 25% DW lignin relative to the total lignocellulosic content. The lignin content (%DW) in lignocellulosic biomass (LB) was calculated with the equation used by Thomsen et al. (2014), where LB is composed of cellulose (${X}_{C}$), hemicellulose (${X}_{H}$) and lignin (${X}_{L}$) (Eqs. 1 and 2).

1 $$LB=\left({X}_{C}+{X}_{H}+{X}_{L}\right)$$

2 $$Lignincontent\left(\%DW\right)=\frac{{X}_{L}\text{*100}}{\text{LB}}$$

Mixed substrates consisted of highly variable biomass sources that did not show any pattern of chemical predominance. For instance, the chemical compositions of food waste and sewage are often affected by culture, season, social class and holidays^46^, making it impossible to precisely determine their chemical composition over time.

### Data analysis

We applied the standardized mean difference (SMD) estimated by Hedge’s g as the effect size with which to quantify methane yield data. Following the formula^27^:

3 $$g=\frac{{Mean}_{T}-{Mean}_{C}}{\sqrt{({n}_{1}-{1)s}_{1}^{2}+({n}_{2}-1){s}_{2}^{2}}}* 1- \frac{4}{4({n}_{1}+{n}_{2})-9}$$

where the ${Mean}_{T}$ is the treated group and ${Mean}_{C}$ is the control group, ${n}_{1}$ and ${n}_{2}$ are the sample size while ${s}_{1}^{2}$ and ${s}_{2}^{2}$ are the estimated population variance for the treated and control group, respectively^27^. This effect size is considered less biased than other calculation approaches and is recommended for small sample sizes^48^.

Mean effect sizes (Hedges’ g), 95% confidence interval (CI) with bias correction and p-value were calculated in R software (R Core Team, 2021) using the “metafor” package (version 3.0-2) for each pretreatment as well as for the specific techniques of significant pretreatment methods^27,49^. Pretreatments were considered significant (p < 0.05) when their mean value and CI did not overlap the zero line. Mean and CI values below the zero line indicated a negative response (pretreatment < control), while mean and CI values above the zero line indicated a positive response (pretreatment > control).

A multilevel meta-analysis was performed followed by a subgroup analysis as the data were grouped into pretreatment categories for analysis^49^. Also, the dependence of effect sizes was considered since a given study can compare several treatments to a single control group, which means that the data are not independent. Furthermore, we assumed the random effect model considering the difference in methodology of experiments performed in each study included in the analysis^27,49^.

### Supplementary Information

Supplementary Information.

Supplementary Table S1.

## Data Availability

The data supporting the findings of this study are publicly available in the Zenodo with the following 10.5281/zenodo.6619882.

## Code availability

The software used for analysis is available from https://www.r-project.org/. The source code is accessible in the tutorial by Assink and Wibbelink^49^.
