# Supplementary material for: Methane yield response to pretreatment is dependent on substrate chemical composition: a meta-analysis on anaerobic digestion systems
Source: Sci Rep. 2024 Jan 12;14:1240. doi: 10.1038/s41598-024-51603-9 (PMC10786828; doi:10.1038/s41598-024-51603-9)
Supplement: Supplementary file 1 — Supplementary Information. [file 41598_2024_51603_MOESM1_ESM.pdf]

Supplementary information for

Methane yield response to pretreatment is dependent on substrate chemical composition: A meta-analysis on anaerobic digestion systems

Thuane Mendes Anacleto, Betina Kozlowsky-Suzuki, Annika Björn, Sepehr Shakeri Yekta, Laura Shizue Moriga Masuda, Vinícius Peruzzi de Oliveira, Alex Enrich-Prast

---

Supplementary Figures

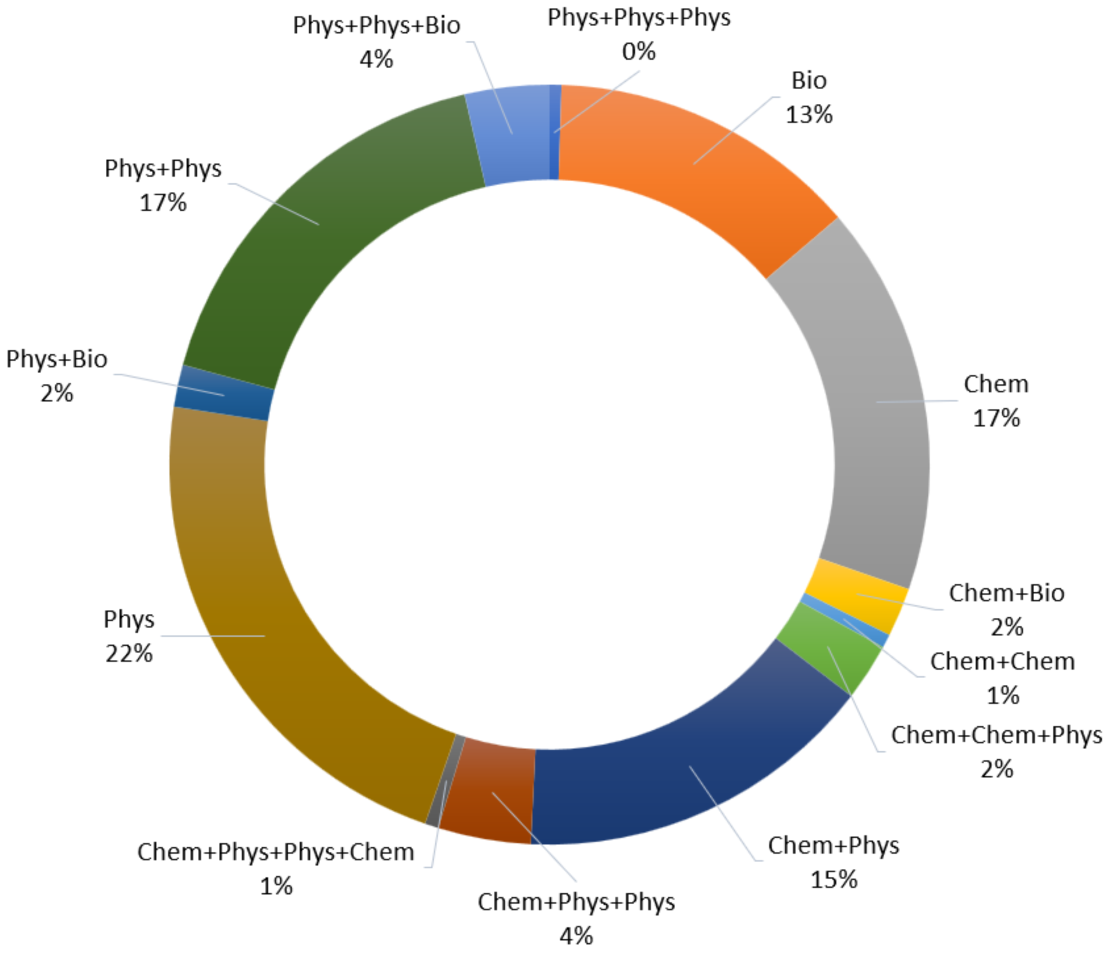

Fig. S1. Quantification in percent of single and combined pretreatments of different nature (i.e., Biological = Bio, Chemical = Chem and Physical = Phys) applied prior to AD from the studies (n = 415) included in this systematic review and listed in Table S5. Detailed information about the different pretreatments can be found in Fig. S2-S4 and Tables S1-S4.

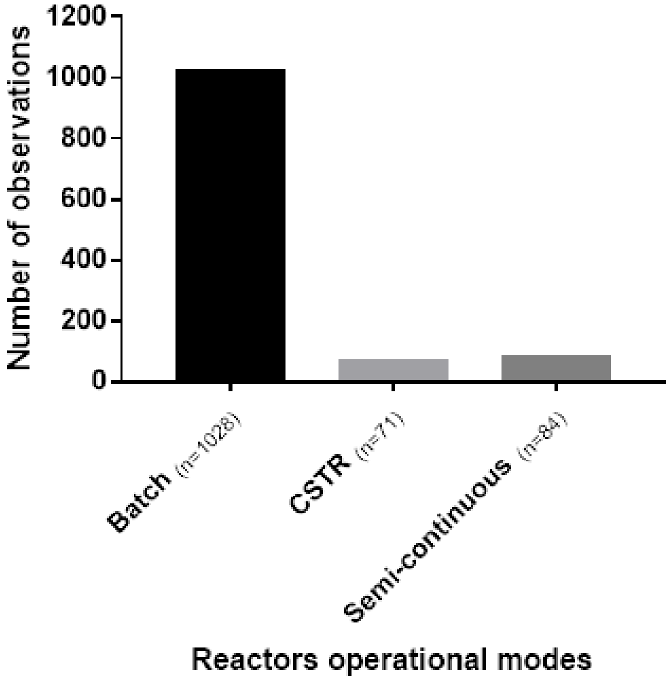

Fig. S2. Summary of reactor operational model used in the studies included in this meta-analysis. The majority of observations were retrieved from studies applying batch incubations, followed by semi continuous feeding and by CSTR: Continuous stirred-tank reactor model.

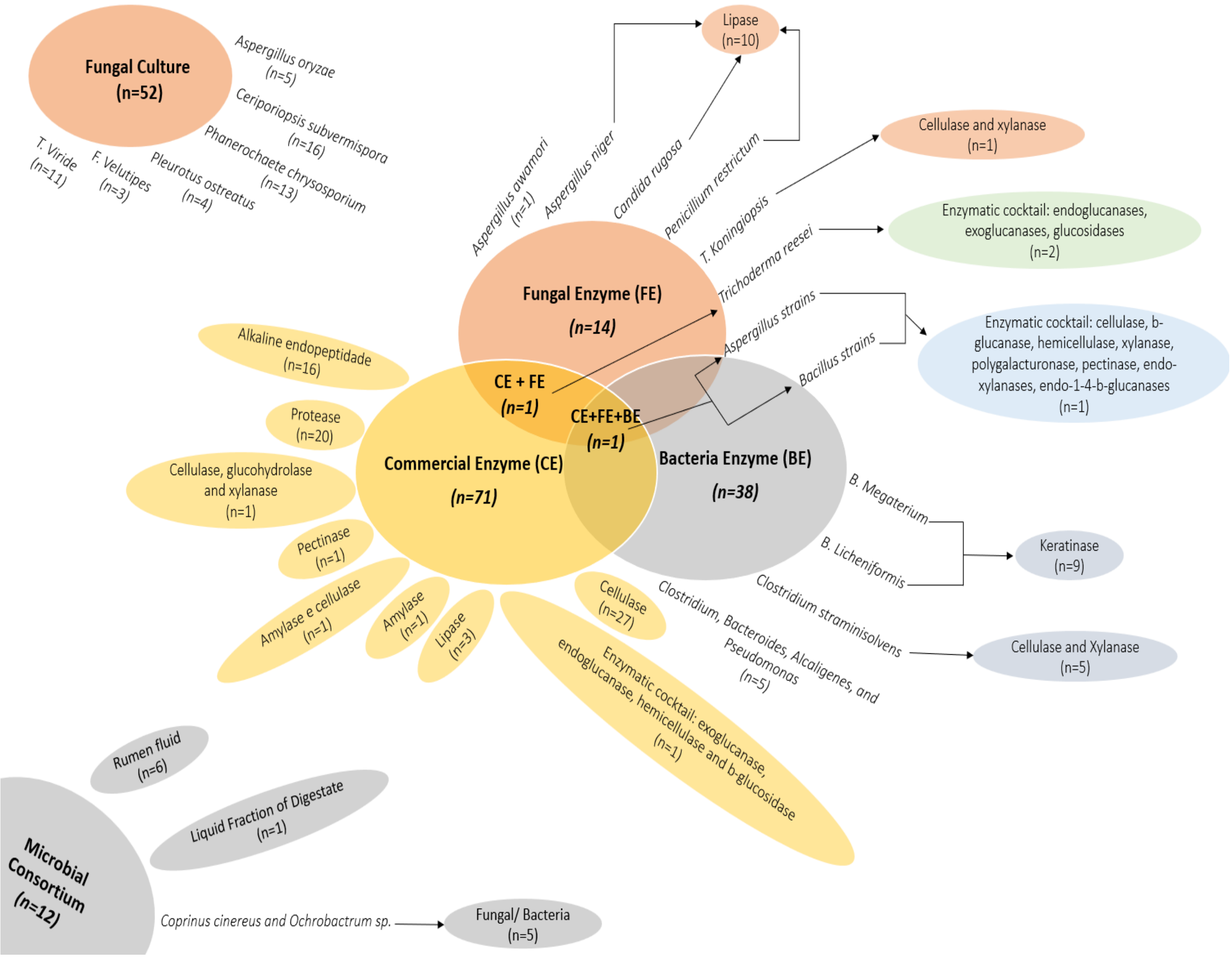

Fig. S3. Overview of the different biological pretreatments reported from the studies included in this meta-analysis. The largest share of cases applied Commercial Enzyme (n = 71), followed by Fungal Culture (n = 52) and Bacterial Enzyme (n = 38). The application of Fungal Enzymes (n = 14) and Microbial Consortium (n = 12) were the least used pretreatments.

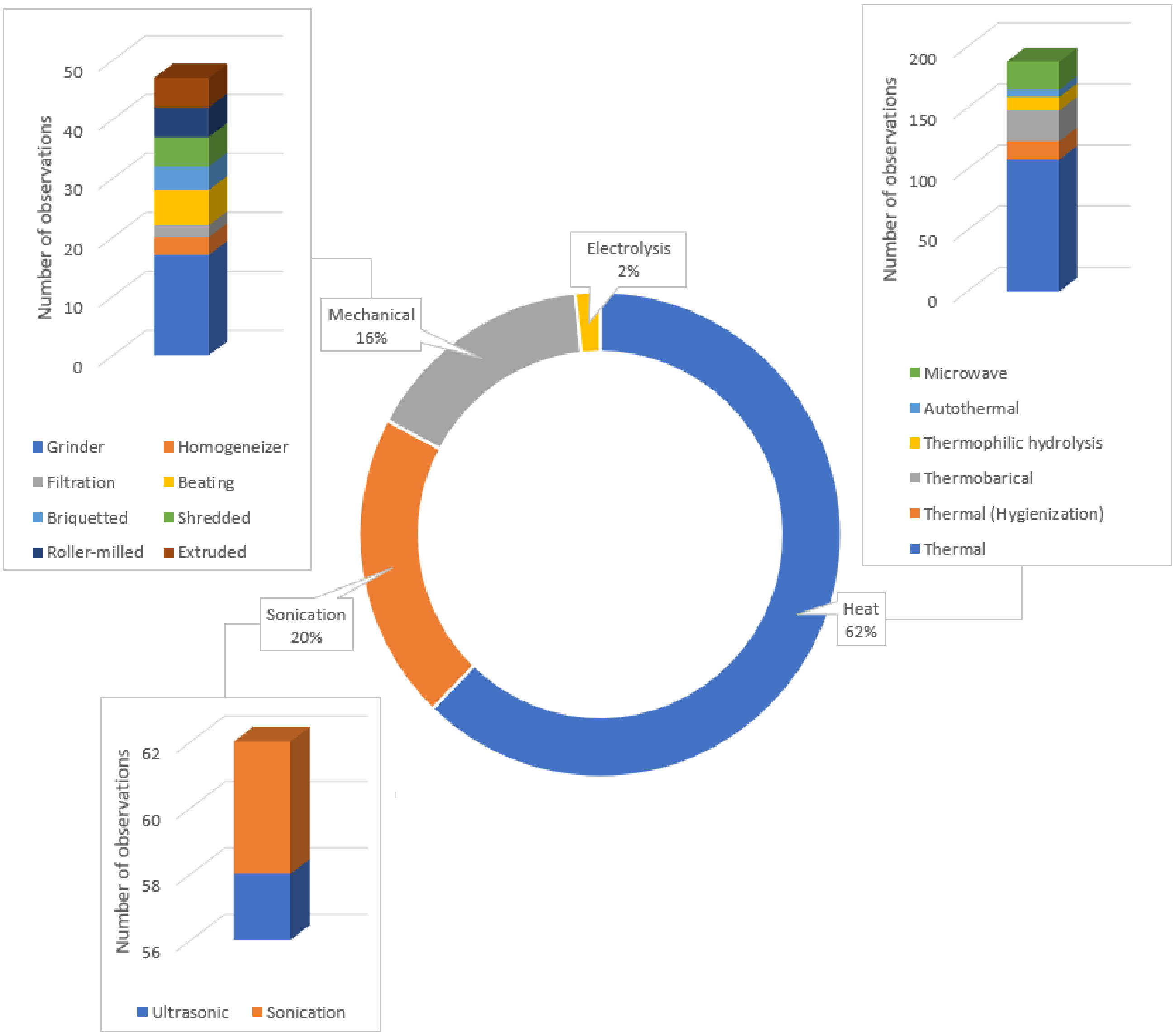

Fig. S4. Overview of the different physical pretreatments reported from the studies included in this meta-analysis. Heat is by far the most common physical pretreatment, followed by mechanical methods where grinder prevails and by sonication. Electrolysis was applied in only 2% of the reported cases.

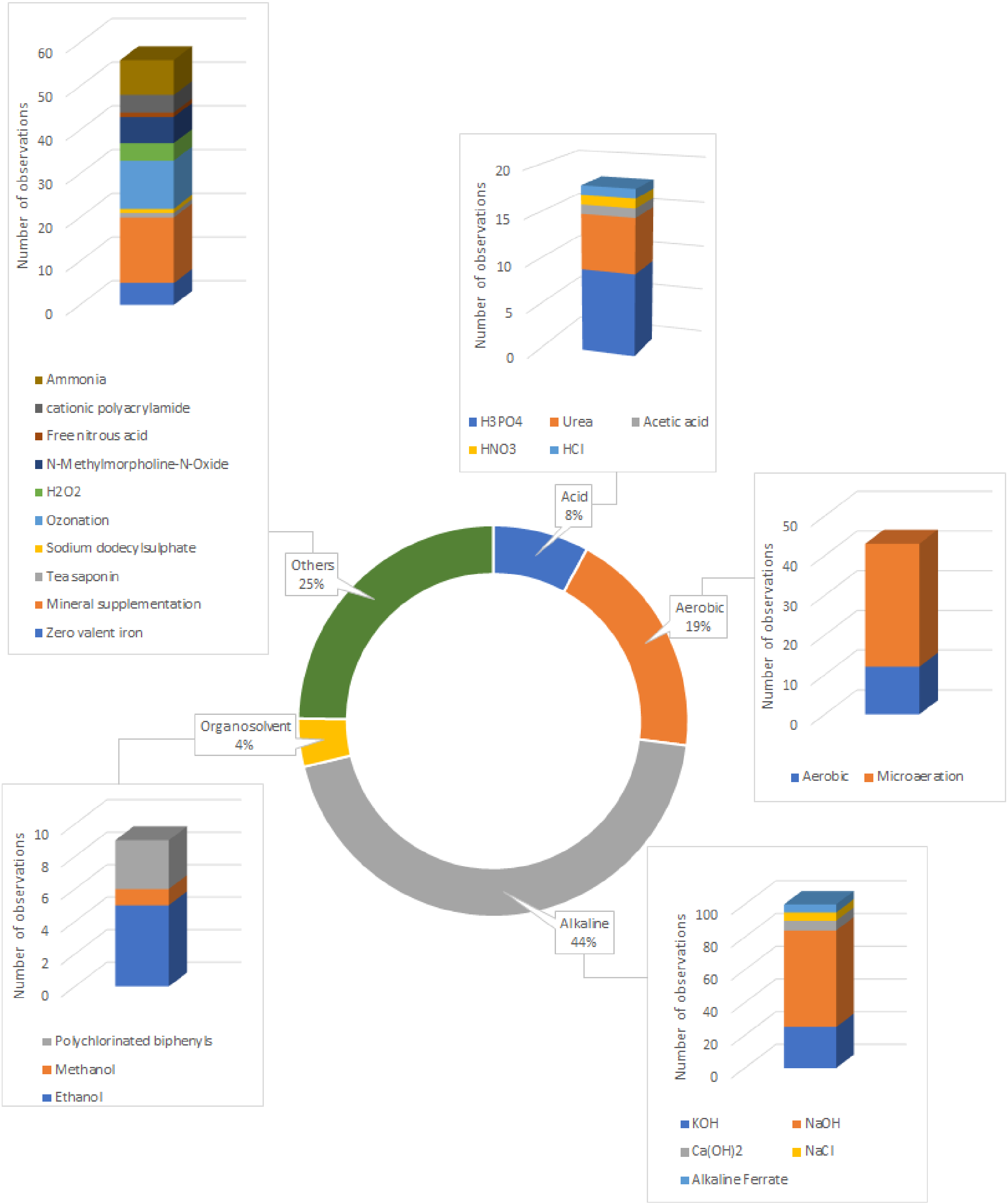

Fig. S5. Overview of the different chemical pretreatments reported from the studies included in this meta-analysis. Alkaline methods responded for 44% of the reported cases. Mineral supplementation, ozonation and ammonia removal were among the most applied methods within the category Others with 25% of the studied cases. Among aerobic methods microaeration prevails, while ethanol is the most applied organosolvent.

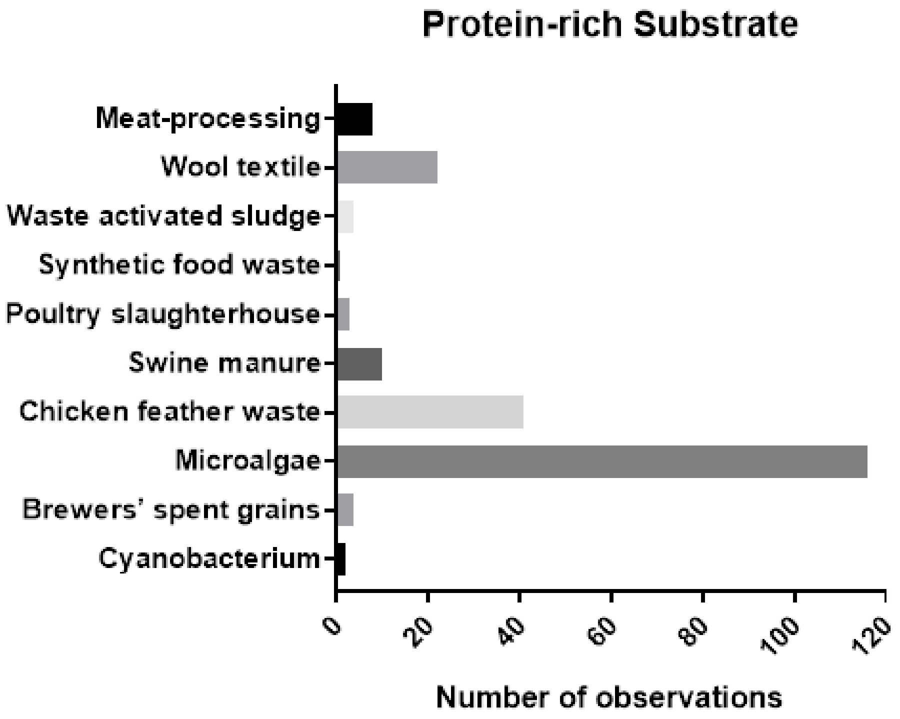

Fig. S6. Biomass sources of the protein-rich substrate. Protein-rich substrate is the substrate of any source with majority in protein (>40% dry matter) such as microalgae and different sorts of animal waste.

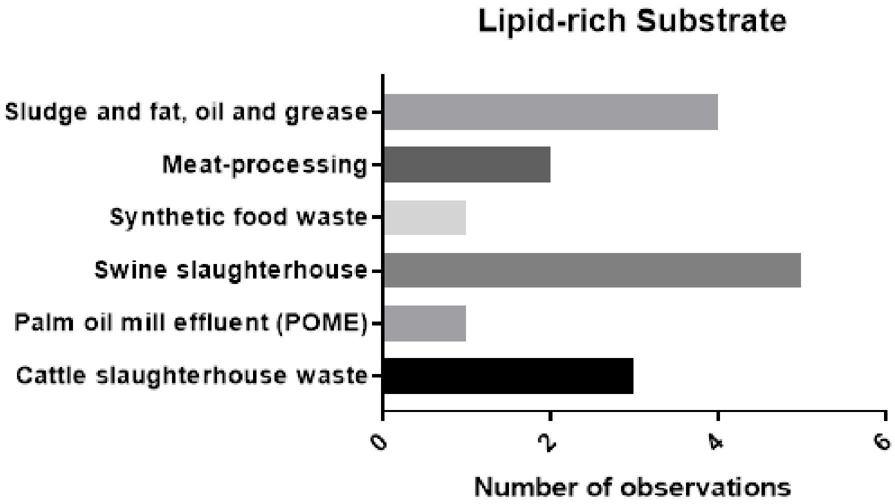

Fig. S7. Biomass sources of the lipid-rich substrate. Lipid-rich substrate is predominantly composed by agricultural oil residues and swine slaughterhouse wastewater. This category of substrate had the lowest number of cases (n = 13) reported.

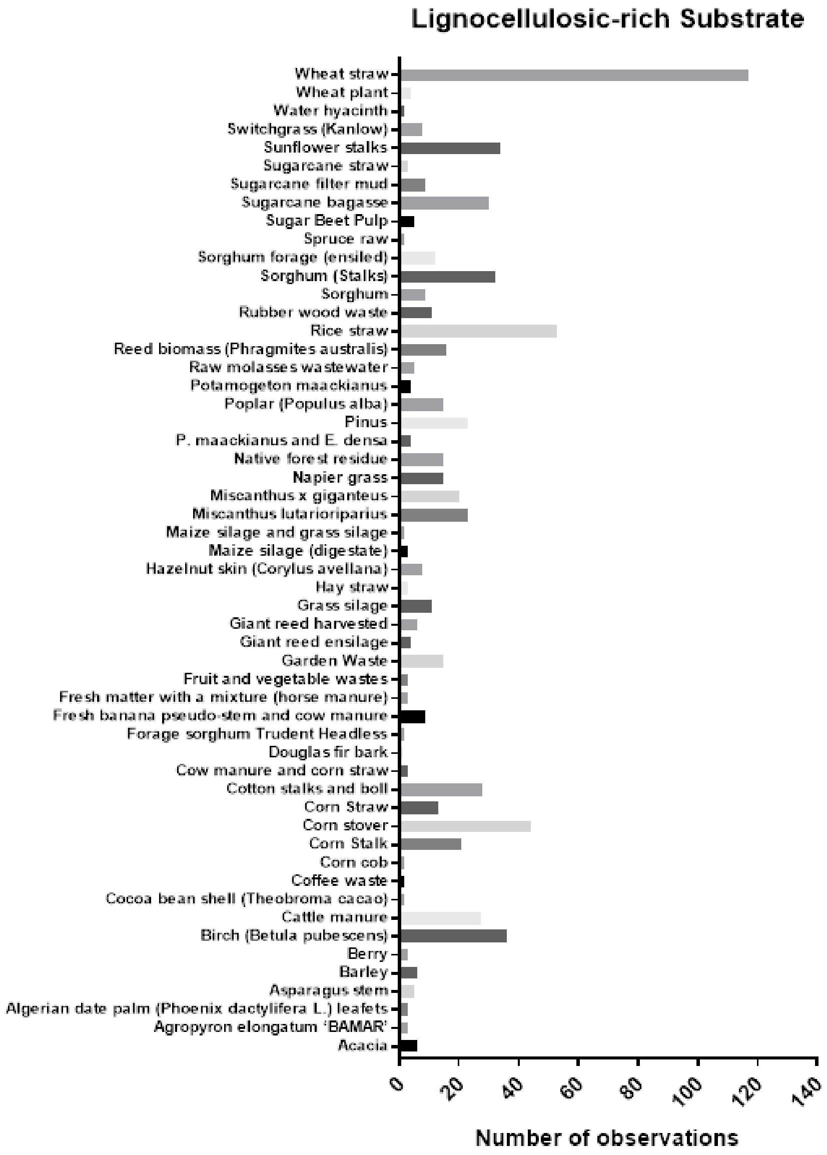

Fig. S8. Biomass sources of the lignocellulosic-rich substrate. This category, predominantly composed by crop residues and cattle manure, was by far the one with the highest number of observations (n = 745).

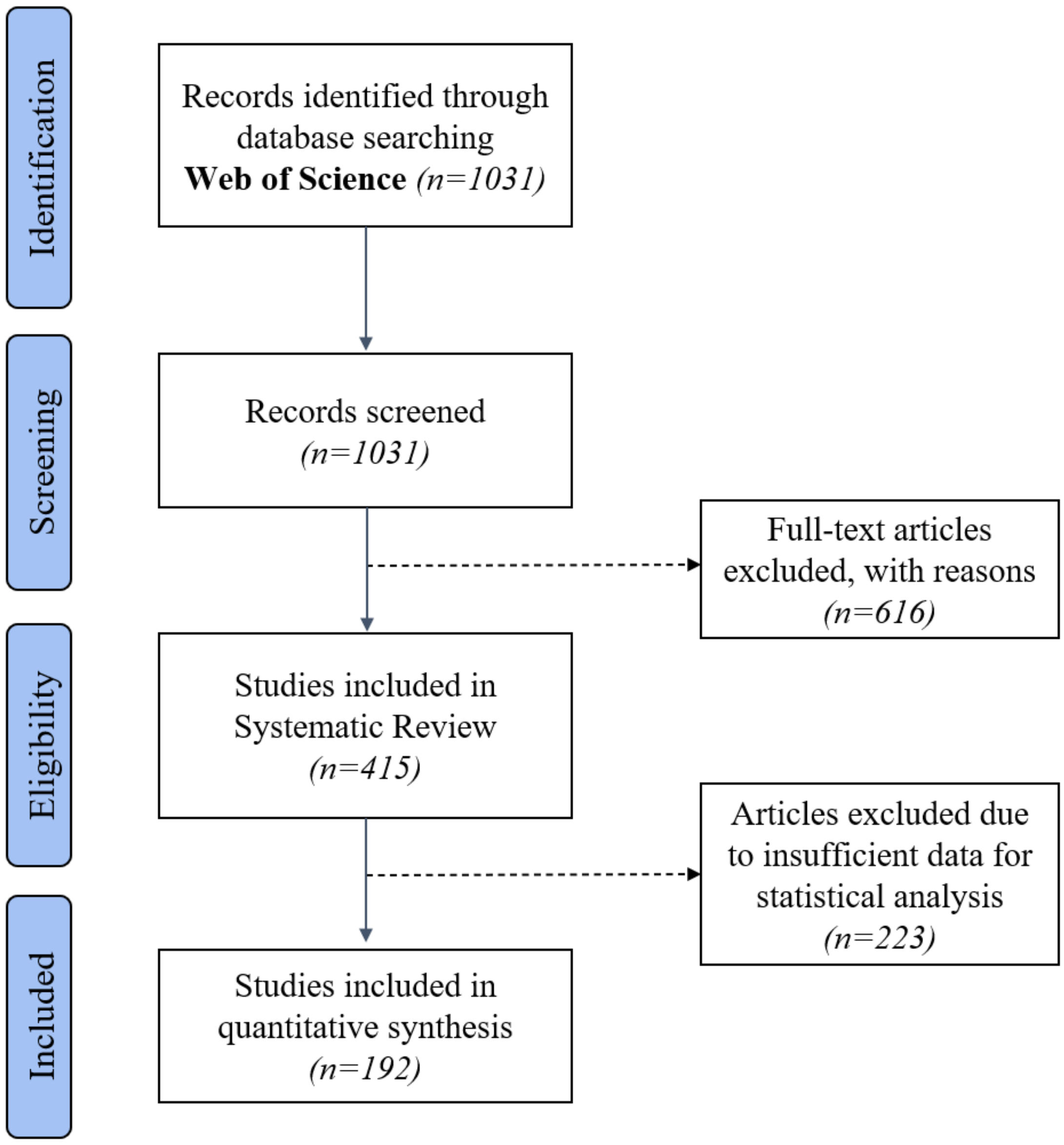

Fig. S9. Flow diagram summarizing quantitatively the selection of studies from the systematic review following the Preferred Reporting Items for Systematic Reviews and Meta-analysis (PRISMA, http://www.prisma-statement.org/). n= number of articles

Supplementary Tables

Table S1. Studies included in the meta-analysis (attached in excel).

Table S2. Performance of the most efficient pretreatments for protein-rich substrate and maximum increase in CH_4 production under specific pretreatment configurations. ($\overline{X} = average$)

	CH_4 Yield [a] $\overline{X}$ Untreated	$\overline{X}$ Pretreated	Change (%)	Specific pretreatment configuration with the maximum increase in CH_4 yield (%)[b]
**Biological**				
Bacterial Keratinase (n=9)	180	270	**50**	↑ 128% From *B. megaterium*, 8 days of degradation [148]
Cellulase (n=2)	253.13	374.34	**47.8**	↑ 72% Concentrated enzyme-substrate, pH 7, for 24 h [58], [158]
Endopeptidase (n=16)	23.8	244.5	**927.3**	↑ 2163% Alkaline endopeptidase 2 h [88], [122]
Enzyme Mix (n=1)	188.6	217.3	**15.2**	↑ 15% Enzyme mix (cellulase, glucohydrolase and xylanase) was added (0.5 and 1% w/w) [58]
Lipase (n=2)	410	624.5	**52.3**	↑ 106% From fungus *Penicillium restrictum* [130]
Protease (n=12)	131.9	260.9	**97.8**	↑ 129% Enzyme Alkaline Serine Protease (Savinase 16 L, Type EX, Novozymes, Denmark) [132]
Saccharified (n=1)	255.35	373.03	**46**	↑ 46% Buffer solution (50 mM sodium citrate and pH 4.8 + Celluclast 1.5 L and Novozyme® 188 were added at 35 filter-paper unit (FPU) and 61.5 cellobiose activity units (CBU) in each reactor, respectively at 50 °C [96]
**Physical+Physical**				
Autoclave (n=13)	196.2	232.9	**18.7**	↑ 1005% 120 °C for 10 min [12], [109], [119], [122], [132]
Hydrothermal (n=6)	102	182.3	**78.8**	↑ 309% 0.82 MPa 200 °C for 0 min [89], [160]
Steam explosion (n=26)	263.6	314.8	**19.4**	↑ 62% 110 ± 5 °C (1.0 ± 0.2 bar) [104], [120], [121], [153]
Thermal+Pressure (Sterilized) (n=1)	580	960	**65.5**	↑ 65% 133 °C and 3 bars for 20 min [131]
Ultrasonication + Electrolysis (n=1)	138	257	**86.2**	↑ 86% Ultrasonication 30 W + Electrolysis 30 V 10 min [49]
**Physical**				
Electrolysis (n=3)	138	123.6	**-10.4**	↑ 31% Electrolysis 30 V 10 min [49]
Homogeneizer (n=3)	317.66	407	**28.1**	↑ 39% Homogeneizer: 220 W and 30 min [153]
Microwave (n=6)	307.6	360.6	**17.3**	↑ 59% 900 W of output power and 3 min [85], [108]
Sonication (n=40)	242.7	282.2	**16.2**	↑ 113% Ultrasonication 30 W 10 min [49], [98], [120], [121], [153]
Thermal (n=12)	276	285.9	**3.5**	↑ 50% Frozen 20 °C [8], [119], [131], [136], [153]
**Physical+Physical +Biological**				
Autoclave + Alkaline serine protease (n=12)	110	234.1	**112.8**	↑ 475% 0.53 mL/g VS enzyme concentration + 120 °C for 10 min [132]

Autoclave+Lipase from *C. rugosa* (n=2)	731.5	786	**7.4**	↑ 20% 121 °C for 20 min prior to addition of the enzyme [119]

[a] mL/gVS or mL/gCOD.
[b] Reference number of the article in Table S1 shown in square brackets.
n= number of data samples reported.

Table S3. Performance of the most appropriate pretreatments for lignocellulosic-rich substrates (lignin<10% DW) and maximum increase in CH_4 production under specific pretreatment configurations. ($\overline{X} = average$)

	CH_4 Yield [a]			
	$\overline{X}$ Untreated	$\overline{X}$ Pretreated	Change (%)	Specific pretreatment configuration with the maximum increase in CH_4 yield (%)[b]
**Biological**				
Mix Bacteria (n=5)	185	214.8	**16.1**	↑ 33% Bacteria (*Clostridium, Bacteroides, Alcaligenes, and Pseudomonas*) 13 days [95]
Cellulolytic bacteria (n=5)	185	212.4	**14.8**	↑ 27% Cellulolytic bacteria (*Clostridium straminisolvens*) 13 days [95]
Mix Fungal (n=1)	277.3	351.4	**26.7**	↑ 27% Enzyme (Mixture of Celustar XL and Agropect pomace (3:1) from *Trichoderma longibrachiatum* (fungi) [10]
Fungal (n=3)	125.75	150.69	**19.8**	↑ 34% Fungal (*F. velutipes*) [63]
Fungi and bacteria mixing (n=5)	185	253.4	**36.9**	↑ 50% Fungi and bacteria mixing (*Coprinus cinereus* and *Ochrobactrum sp.*, respectively) [95]
Rumen fluid (n=6)	156.1	238.5	**52.7**	↑ 83% 24 h of pretreatment rumen fluid from the fresh stomach of cattles [30]
**Chemical+Physical**				
Briquetted + Alkali (n=8)	309.15	335	**8.3**	↑ 14% Briquetted + Injected concentration KOH 6,27% (w/w) [31]
Alkali+Thermal (n=7)	152	171.4	**12.7**	↑ 43% 40 °C at 1 h 10 (% w/w) NaOH dosage [116], [117]
Ultrasonic+Alkaline (n=2)	187	275.5	**47.3**	↑ 71% Dual-frequency (20 KHz and 57 KHz) ultrasonic for 30 min + (2% NaOH) for 36 h [169]
**Physical+Physical+Biological**				
Autoclave + Enzyme (n=1)	277.3	534.3	**92.6**	↑ 93% 120 °C, 4 bars for 15 min + Mixture of Celustar XL and Agropect pomace (3:1) [10]

[a] mL/gVS or mL/gCOD.
[b] Reference number of the article in Table S1 shown in square brackets.
n= number of data samples reported.

Table S4. Performance of the most appropriate pretreatments for lignocellulosic-rich substrates (lignin10 – 25% DW) and maximum increase in CH_4 production under specific pretreatment configuration. ($\overline{X} = average$)

	CH_4 Yield [a]			
	$\overline{X}$ Untreated	$\overline{X}$ Pretreated	Change (%)	Specific pretreatment configuration with the maximum increase in CH_4 yield (%)[b]
**Biological**				
Fungal (n=16)	136.2	230.8	**69.4**	↑ 231% Fungal strain (*Phanerochaete chrysosposrium*) [4], [13], [187]
Cellulolytic enzyme (n=4)	146.5	166.2	**13.4**	↑ 36% 1% Cellulolytic enzyme [88]
Microbes (n=1)	174.3	233.3	**33.8**	↑ 34% 2% of liquid fraction of digestate [26]
Saccharified (n=2)	271.9	366	**34.6**	↑ 38% 30 mL citratre buffer solution (50 mM sodium

				citrate and pH 4.8 at 50°C) [96]
**Chemical**				
Alkali (n=28)	27.6	22	**-20.2**	↑ 273% 7% NaOH 50g/L [59], [76], [83], [102]
Acid (n=3)	100.7	128.9	**28**	↓ 11%8 mL of 85% phosphoric acid at 60 °C for 45 min in 50 mL plastic centrifuge tube [40]
Urea (n=2)	210.4	289.4	**37.5**	↑ 45%1% Urea [52]
Ammonia (n=1)	174.3	214.5	**23**	↑ 23% 2% ammonia solution [26]
Microaeration (n=4)	152	234.2	**54**	↑ 82% Aeration time: 48 h; equivalente aerated O_2 intensity (mL O_2/gVS): 431 [29]
Nutrient (n=4)	146.5	160	**9.2**	↑ 20% Nutrient concentrations for the basal medium (1 g/L substrate, containing inorganic macronutrients) were (mg/L): NH_4Cl (76.4), KH_2PO_4 (5.18), $MgSO_4 \cdot 7H_2O$ (0.27), $CaCl_2 \cdot 2H_2O$, (10.00), and trace nutrients, 1 mL/L [88]
**Chemical+Biological**				
Fungal+Moisture (n=18)	120	179.9	**49.9**	↑ 119% *P. ostreatus* 75% at 20 days [20]
Fungal+Acid (n=4)	120	257.5	**114.5**	↑ 150% *N. intermedia* CBS 131.92 (inoculum) + Phosphoric Acid concentration 1.2% (w/v), residence time 7 min, and temperature 195 ± 2 °C [141]
Bacterium+Microaeration (n=5)	230.2	256	**11.2**	↑ 17% 0.10 U/mL min after 24 h bacteria microaerobic pretreatment 1 (v/v, biogas liquid/bacteria solution (*Bacillus Subtilis*) [54]
CaO + Liquid Fraction of Digestate (microbes) (n=1)	174.3	274.6	**57.4**	↑ 57% 6% CaO + Liquid Fraction of Digestate (LFD) [26]
**Chemical+Physical**				
Acid + Mold Size (n=2)	213	223	**4.7**	↑ 3% Acetic acid (HAc) 1% + 68 mm [64]
Size reduction + Alkali (n=16)	274.5	261.7	**-4.6**	↑ 9% KOH 1% + 68 mm [64], [27]
Alkaline + Thermal (n=1)	122.3	173.3	**41.7**	↑ 41% NaOH (0.25 N, temp. 50 ºC, incubation time 30 min) [72]
Acid + thermal (n=9)	160.8	176.1	**9.5**	↑ 22% Furfural acid + 6 days 35 °C [186]
Thermal + Organosolv (n=18)	92.9	172.7	**85.9**	↑ 270% 160 ºC + 50% ethanol [81], [156]
**Chemical+Physical+Physical**				
Lewis acids organosolv catalysed FeCl2 + Autoclave (n=6)	189	294.6	**55.8**	↑ 61% H_2SO_4 (40 g of WS was mixed with 1.25 L of aqueous ethanol (EtOH 65%, H_2O 35%) with 8 mmol L1 Lewis acid (or 4.4 mmol L1 H_2SO_4) in a 2 L autoclave during 2 h at 160 ºC [45]
**Physical**				
Mechanical (Size reduction) (n=28)	154.8	175.6	**13.4**	↑ 55% The extruder barrel is 2.84 m long and the retention time for the biomass varied from 37 to 82 s. Feeding was done at 528 rpm and the extrusion screws ran at 600 rpm [13], [27], [42], [64]
Thermal (n=12)	316.4	411.1	**29.9**	↑ 70% 35 °C for 6 days [9], [23], [178]
**Physical+Biological**				
Milling + Fungal (n=6)	97	217.6	**124.3**	↑ 166% Fungus *Pleurotus ostreatus* (DSM 11191) 20 days + Milling (<2 mm) [13]
**Physical+Physical**				
Autoclave (n=7)	219.1	231.5	**5.6**	↑ 18% 121 °C for 30 min [4], [13]
Hydrothermal (n=18)	173	178	**2.9**	↑ 69% 175 °C at 30 min [1], [191]
Steam explosion (n=44)	239.3	264.4	**10.5**	↑ 89% 200 °C for 15 min [15], [114]
Filtration +	261	228.	**-12.6**	↓ 5% Sieved fresh incoculum stored at 4 °C in cold room

Temperature (n=2)		prior to batch tests [23]

[a] mL/gVS or mL/gCOD.
[b] Reference number of the article in Table S1 shown in square brackets.
n= number of data samples reported.

Table S5. Performance of the most appropriate pretreatments for lignocellulosic-rich substrates (lignin content >25% DW) and maximum increase in CH_4 production under specific pretreatment configuration. ($\overline{X} = average$)

	**CH_4 Yield [a]**			
	$\overline{X}$ **Untreated**	$\overline{X}$ **Pretreated**	**Change (%)**	**Specific pretreatment configuration with the maximum increase in CH_4 yield (%)[b]**
**Chemical**				
Acid (n=5)	166.32	254.9	**53.2**	↑ 560% Acetic acid (without catalyst) [90], [192]
Alkali (n=10)	235.7	250.8	**6.4**	↑ 132% 12% NaOH [19], [92], [93], [189]
Micro-aeration (n=15)	257	261.3	**1.6**	↑ 7% 5 mL O_2/g VS, Pulse: 1 for 3 days [17]
Organosolv (n=2)	50	265	**430**	↑ 500% Ethanol (without catalyst) [90]
Urea (n=4)	157.5	164.2	**4.2**	↑ 7% Urea 2% 60 day [75]
**Chemical+Chemical**				
Organosolv+ Acid (n=4)	50	325	**550**	↑ 580% Methanol+Sulfuric Acid/ Ethanol and Sulfuric Acid [90]
Acid + Acid (n=2)	50	295	**490**	↑ 520% Acetic acid and Sufuric Acid [90]
**Chemical+Chemical+Physical+Physical**				
Hydrothermal + Organosolv + Acid (n=8)	124	239.1	**92.8**	↑ 123% 160 °C for 30 min and H_2SO_4 with 1% w/w and isopropanol [56]

[a] mL/gVS or mL/gCOD.
[b] Reference number of the article in Table S1 shown in square brackets.
n= number of data samples reported.
